# Change in physical activity and systolic blood pressure trajectories throughout mid-life and the development of dementia in older age: the HUNT study

**DOI:** 10.1186/s11556-023-00328-1

**Published:** 2023-10-02

**Authors:** Maren Lerfald, Stian Lydersen, Ekaterina Zotcheva, Tom I. L. Nilsen, Rannveig S. Eldholm, Nicolas Martinez-Velilla, Geir Selbæk, Linda Ernstsen

**Affiliations:** 1 Department of Public Health and Nursing, Faculty of Medicine and Health Science, Norwegian University of Science and Technology, PO box 8950, N-7491 Trondheim, Norway; 2 Clinic of Medicine, St. Olavs Hospital, Trondheim University Hospital, Trondheim, Norway; 3 Department of Mental Health, Faculty of Medicine and Health Science, Norwegian University of Science and Technology, Trondheim, Norway; 4 Norwegian National Centre for Ageing and Health, Vestfold Hospital Trust, Oslo, Norway; 5 Clinic of Anaesthesia and Intensive Care, St. Olavs Hospital, Trondheim University Hospital, Trondheim, Norway; 6 Department of Geriatrics, Clinic of Medicine, St.Olavs Hospital, Trondheim, Norway; 7 Department of Neuromedicine and Movement Science, Faculty of Medicine and Health Science, Norwegian University of Science and Technology, Trondheim, Norway; 8 Navarrabiomed, Hospital Universitario de Navarra, UPNA, IdiSNA, Pamplona, Spain; 9 Faculty of Medicine, University of Oslo, Oslo, Norway; 10 Department of Geriatric Medicine, Oslo University Hospital, Oslo, Norway

**Keywords:** Physical activity, Blood pressure, Trajectories, Dementia

## Abstract

**Background:**

There is lack of research on combinations of possible modifiable risk factors for dementia in a life-time perspective. Dementia has currently no cure, and therefore new knowledge of preventive factors is important. The purpose of this study is to investigate if changes in physical activity (PA) in combinations with systolic blood pressure (SBP) trajectories in mid to late life are related to development of dementia in older age.

**Methods:**

This prospective cohort study uses data from four consecutive surveys of the HUNT Study, Norway. Dementia was assessed in the HUNT4 70 + sub-study (2017–19). Group-based trajectory modelling identified three SBP trajectories from HUNT1 (1984–86) to HUNT3 (2006–2008): low, middle, and high. Change in PA was categorized into four groups based on high or low PA level at HUNT1 and HUNT3 and were combined with the SBP trajectories resulting in 12 distinct categories. Logistic regression was used to estimate odds ratios (ORs) of dementia.

**Results:**

A total of 8487 participants (55% women, mean age (SD) 44.8 (6.5) years at HUNT1) were included. At HUNT4 70 + , 15.2% had dementia. We observed an overall decrease in OR of dementia across the PA/SBP categories when ranked from low to high PA (OR, 0.96; 95% CI, 0.93 to 1.00, *P* = 0.04). Within PA groups, a low SBP trajectory was associated with lower OR for dementia, apart from those with decreasing PA. The strongest association was observed for people with stable high PA and low SBP trajectory (OR, 0.38; 95% confidence interval (CI), 0.13 to 1.10 and adjusted risk difference, -8.34 percentage points; 95% CI, -15.32 to -1.36).

**Conclusion:**

Our findings illustrate the clinical importance of PA and SBP for dementia prevention and that favorable levels of both are associated with reduced occurrence of dementia.

**Supplementary Information:**

The online version contains supplementary material available at 10.1186/s11556-023-00328-1.

## Introduction

The incidence of dementia seems to be decreasing [1] but due to demographical changes with an increasing older population the number of people living with dementia is increasing [2]. A recent publication estimated an increase in the prevalence of dementia from 57 million individuals in 2019 to 153 million individuals worldwide in 2050 [3]. In Norway, this number is estimated to increase from 101 thousand individuals in 2020 to 237 thousand in 2050 [4]. A report from the Lancet Commission on dementia prevention, intervention and care identified 12 modifiable risk factors, including physical activity (PA) and blood pressure (BP), and estimated that targeting these might delay or prevent 40% of all dementia cases [2].

Physical activity has been shown to reduce dementia risk in recent studies [5–7]. Although PA is among the suggested modifiable risk factors to prevent dementia [2, 8–11], the limitations of evidence have recently been emphasized [12]. The effect of PA on dementia follows different physiological, cellular, and molecular mechanisms [10, 13, 14]. A recent meta-analysis found timing of PA assessment to be crucial for risk estimation [15]. PA assessed 10 years or more before diagnosis was not associated with dementia risk, whereas PA assessed less than 10 years before diagnosis, showed higher risk among the inactive, possibly due to reverse causation. Some studies note that higher intensity levels of PA are crucial for prevention [11, 16]. The complexity of the relation between PA and dementia calls for further investigation [17].

Previous studies have found increased systolic BP (SBP) in mid-life to be associated with higher dementia risk in older age [18–21]. A recent systematic review of 209 studies examining the association between BP and dementia risk or cognitive impairment reported an overall stronger association of BP in mid-life compared to late-life [22]. Five studies included in dose–response analyses indicated mid-life SBP > 130 mmHg to be associated with higher risk of cognitive disorders [22]. Higher SBP in mid-life, and a decrease in SBP prior to dementia diagnosis was found when investigating SBP trajectories among people with dementia, compared to those without dementia [19]. In late-life, an association between lower BP and increased dementia risk has been reported [19, 23, 24].

Directions for dementia prevention research emphasize lack of knowledge on lifetime exposure patterns, combinations of risk factors and precision in exposure measures [25]. Change in PA and SBP before a dementia diagnosis could be due to reverse causation meaning that the change is happening due to neurodegenerative processes rather than causing them. To investigate this, studies with long follow-up and repeated measurements are needed. In addition, increasing modifiable risk factor knowledge on dementia is of great importance for both clinical practice and public health [26].

Therefore, the aim of our study is to investigate the joint association of long-term changes in PA and temporal trajectories in SBP over 24 years on the development of dementia in older age.

## Methods

### Study population

This study uses data from the population-based HUNT Study in Norway. The entire adult population in the geographic region of North Trøndelag has been invited to participate in clinical examinations, interviews, questionaries, laboratory measurements and to provide biological samples in four consecutive surveys: HUNT1 (1984–86), HUNT2 (1995–97), HUNT3 (2006–08) and HUNT4 (2017–19) [27]. The HUNT Study has a high participation rate ranging from 58 to 89% [28]. Details about the HUNT study are described elsewhere [27–30].

**Fig. 1:**
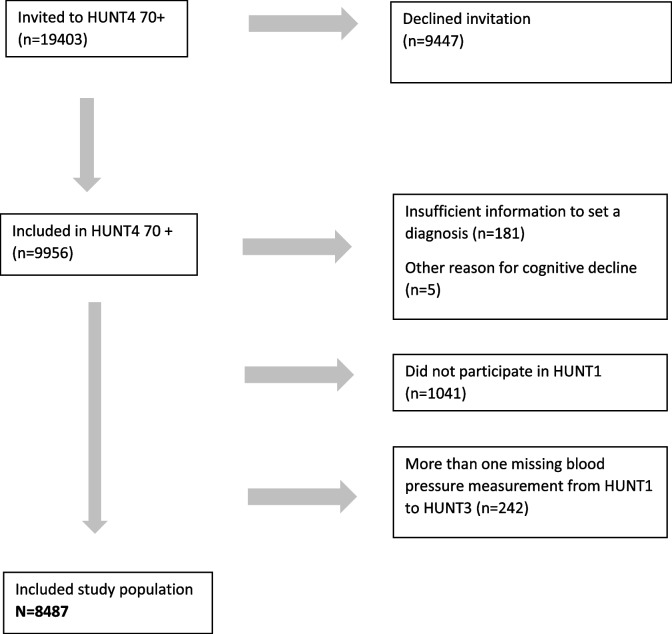
Flow chart of study population

At HUNT4, individuals aged 70 years and older were invited to a sub study called HUNT4 70 + consisting of questionnaires and physical examinations including a clinical cognitive assessment [4]. For the present study, we included those who accepted the invitation (51.3%) and participated in HUNT4 70 + (*n* = 9956), and excluded those who had insufficient information from the cognitive assessment (*n* = 181), other reasons for cognitive decline (*n* = 5), did not participate in HUNT1 (*n* = 1041) or had missing data on mean SBP on more than one survey from HUNT1-3 (*n* = 242) (see Fig. 1). Mean (SD) age at HUNT1 in the study population (*n* = 8487) was 44.8 (6.5) years.

### Physical activity

Weekly PA was assessed by identical validated questionnaires at HUNT1 and HUNT3 [31]. Frequency was assessed by “How often do you exercise?” with the alternatives *“never” “less than once a week”, “once a week”, “2–3 times a week”* and *“nearly every day”*. Duration was assessed by “How long do you exercise each time?” with the alternatives *“less than 15 min”, “15–30 min”, “30–60 min”* and *“more than 60 min”.* Intensity was assessed by “How hard do you exercise?” with the alternatives *“I take it easy, I don’t get out of breath or break into a sweat*”, “I *push myself until I am out of breath and break into a sweat”, and “I practically exhaust myself”.* The last two intensity levels were categorized as moderate and vigorous, respectively. The lowest intensity level is in the lower scale of moderate intensity level, and to reduce the risk of misclassification into moderate intensity, this level was set to below moderate intensity level. The World Health Organization (WHO) recommends at least 150–300 min of moderate-intensity, or 75–150 min of vigorous-intensity PA weekly among adults for their health [32]. To calculate minutes of weekly PA in moderate and vigorous intensity, we multiplied minutes of activity (median duration) with frequency (minutes*frequency) and set a cut-off of high PA to a minimum of 150 or 75 min of moderate or vigorous PA, respectively. PA was grouped into high (meets the recommendations of intensity and duration from WHO) and low (does not meet the recommendations of intensity and duration from WHO) at both HUNT1 and HUNT3 timepoints.

### Systolic blood pressure

SBP was retrieved from HUNT1, HUNT2 and HUNT3. At HUNT1, SBP was measured twice in sitting position with a minimum of five minutes rest between the measurements [23]. Measurement was performed by trained nurses or technicians using a mercury sphygmomanometer. At HUNT2 and HUNT3, BP was automatically measured three times with one-minute intervals using an oscillometry-based device (Critikon Dinamap 845XT and XL9301; GE Medical Systems Information Technologies, Barrington, IL, USA) [23, 29]. In addition, at HUNT2-3, a Dinamap CL model 9301 (Johnson &Johnson Medical Inc.) was used to measure BP by the team who went to the smaller municipalities [19]. Mean SBP was calculated from measure one and two in HUNT1 and from measure two and three in HUNT2 and HUNT3.

### Combined change in PA level and SBP trajectories

**Fig. 2:**
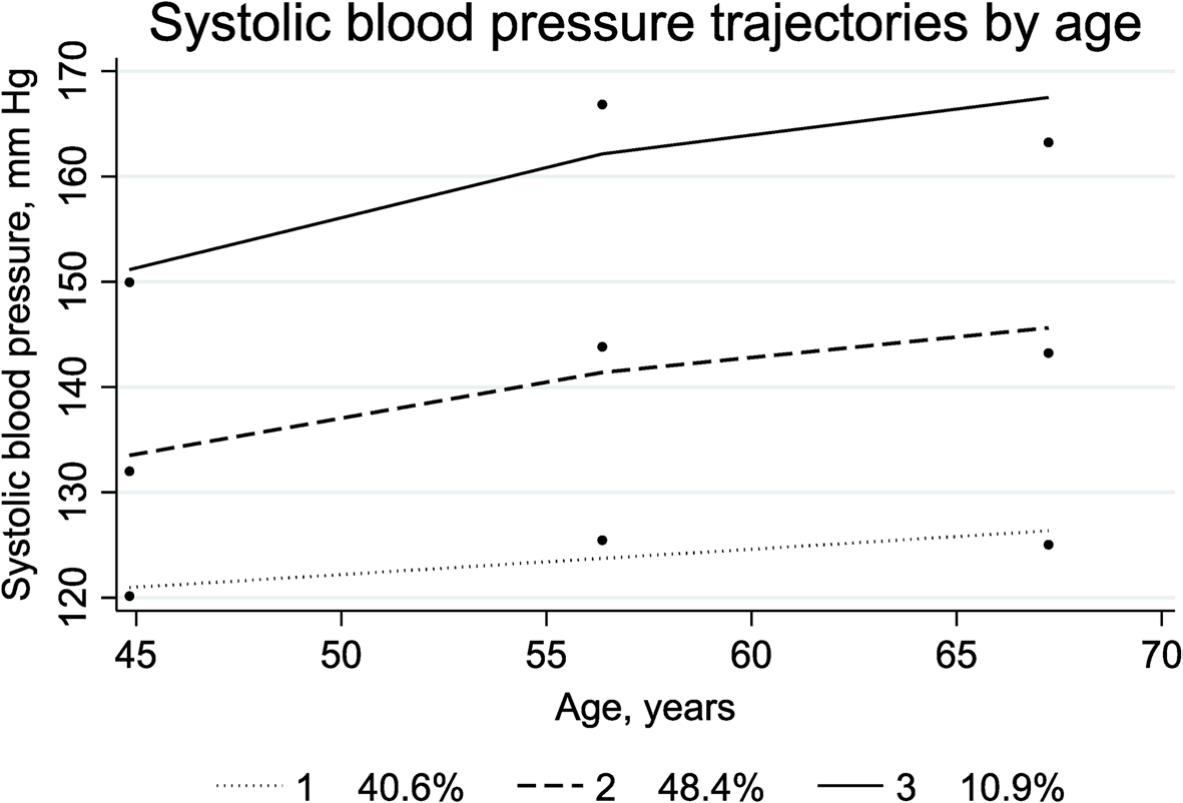
Systolic blood pressure trajectories by age (*n* = 8487) with estimated percentage of the sampled population belonging to the trajectory. Trajectory 1: low trajectory (dotted line); trajectory 2: middle trajectory (dashed line), and trajectory 3: high trajectory (solid line). The dots represent the observed group means

We categorized PA change from HUNT1 to HUNT3 into four groups ordered according to the expected most to least favorable change: stable high, low to high, high to low, and stable low. Group-based trajectory modeling was used to identify individuals following similar SBP patterns across time. Group-based trajectory modeling is a finite mixture model that uses maximum likelihood to estimate model parameters [33, 34]. To get as precise estimates as possible, all included participants had at least two valid BP measurements from HUNT1 to HUNT3 (see Fig. 1). We used the traj package in Stata to perform the trajectory modelling [35]. We chose three groups based on the best Bayesian information criteria (BIC) in combination with our research question (Fig. 2). The model fit was assessed as suggested by Nagin (Additional file 1: Appendix 1 and Appendix 2) [36]. The four PA levels were combined with the three SBP trajectory groups resulting in a joint PA and SBP variable with 12 distinct categories.

### Dementia assessment

The diagnostic evaluation in HUNT4 70 + was based on the DSM-5 diagnostic criteria [37]. Two medical doctors from a pool of nine, with expertise in geriatrics, old-age psychiatry, or neurology, gave an independent diagnosis based on all relevant information including cognitive tests, functioning in daily life, neuropsychiatric symptoms, development of the condition, subjective cognitive decline, and interviews with next-of-kin [4]. Those assigned a dementia diagnosis were categorized into a specific dementia type. More details on cognitive assessment in HUNT4 70 + can be found in Gjøra et al. [4]. In this study, participants were categorized into either all-cause dementia or no dementia.

### Covariates

Potential confounders were assessed at HUNT1 and included age (years), sex (men, women), education (primary, high school, college or university ≤ 4 years, college or university > 4 years), marital status (married or not), smoking (never, former, daily smoker), alcohol use frequency last 14 days (abstainer, did not drink, 1–4 times, more than 5 times), body-mass index (BMI, kg/m^2^), diabetes (yes, no), anxiety and depression index (ADI-4) [38], and Apolipoprotein E ε4 (APOE ε4) status (APOE ε4 carrier or not). For education, we retrieved information from HUNT2 if no information was available at HUNT1. How the genetic information was retrieved and handled is described elsewhere [28]. If the genetic information was indistinct (*n* = 8), the values were recoded as missing.

### Statistical analyses

We used binary logistic regression with dementia as dependent variable and the PA/SBP groups as twelve category independent variable, with stable low PA and high SBP as reference. The first model was adjusted for sex and age, and the second model was fully adjusted for the above listed covariates. Next, we estimated the adjusted absolute risk difference (ARD) compared to the reference category based on the second model [39]. In a similar logistic regression model, the twelve categories were entered as an ordinal variable ranked from stable low PA/high SBP to stable high PA/low SBP and organized by increasing PA, to assess trend across the categories. We report 95% confidence intervals (CI) where relevant. Stata MP version 17.0 (StataCorp LLC, College Station, TX, USA) was used for all analyses.

#### Sensitivity analysis

The main analysis was a complete case analysis including 6156 subjects. To assess potential bias due to missing values we also conducted a sensitivity analysis using multiple imputation in the entire sample of 8478 participants, creating 100 imputed data sets [40], and repeated the logistic regression to assess if this influenced the estimates. The imputation model included all variables in the analysis models, and long-term illness at HUNT1 to HUNT3 was included as auxiliary variable. We also excluded those who reported current or previous use of BP lowering medications (*n* = 405) and those who did not answer the question regarding BP medications (*n* = 1) at HUNT1 and repeated the imputation process and regression analysis.

### Ethics

In all four surveys of the HUNT Study, participants gave informed written consent. The present study was approved by the Regional Committee of Medical and Health Research Ethics in Norway (REK sør-øst D 2017/382). Storage and use of data applies with the General Data Protection Regulation (GDPR).

### Declaration of sources of funding

This work was supported by the Liaison Committee for education, research, and innovation in Central Norway [grant number 2022–30545]. The funding source did not have any role in the design, execution, analysis and interpretation of data, or writing of the study.

## Results

A total of 8487 participants (55.5% women) with a mean age of 44.8 (SD, 6.5 years) years at HUNT1 were included. At HUNT4 70 + , 15.2% had dementia. Of these, 57.8% had Alzheimer’s disease, 10.2% had vascular dementia, 8.9% had mixed dementia and 23.1% had other or unspecified dementia types. Around 80% were in the stable low PA group, and at HUNT3, 15.9% met the criteria for high PA, while 5.8% met the PA criteria at HUNT1. The lowest SBP trajectory contained 39.9% of the sample and started at a SBP level around 120 mm Hg, the middle trajectory contained about 51% of the sample and started at a SBP level a bit higher than 130 mm Hg, and around 10% were assigned to the highest trajectory and started at a SBP level around 150 mm Hg. All three trajectories increased slowly and similarly. Descriptive statistics including missing values are presented in Table 1. Distribution of dementia cases among the 12 different PA/SBP groups can be found in Additional file 1: Appendix 3.

**Table 1:** Descriptive statistics of the study population (*n* = 8487) by systolic blood pressure (SBP) trajectories

	**SBP low trajectory**	**SBP middle trajectory**	**SBP high trajectory**	**Missing, No. (%)**	**Total**
Total study population, No. (%)	3 383 (39.9)	4 293 (50.6)	811 (9.6)	0 (0)	8 487 (100)
Sex, No. (%)				0 (0)	
Women	2 065 (61.0)	2 187 (50.9)	461 (56.8)		4 713 (55.5)
Age^a^, mean (SD)	44.2 (6.1)	44.9 (6.5)	46.8 (7.6)	0 (0)	44.8 (6.5)
Education^a^, No. (%)				121 (1.4)	
i. primary	1 430 (43.0)	2 050 (48.4)	469 (58.5)		3 949 (47.2)
ii. high school	1 234 (37.1)	1 470 (34.7)	256 (31.9)		2 960 (35.4)
iii. college/university < = 4 years	364 (10.9)	408 (9.6)	46 (5.7)		818 (9.8)
iv. college/university > 4 years	301 (9.0)	307 (7.3)	31 (3.9)		639 (7.6)
Marital status^a^, No. (%)				12 (0.1)	
Married	3 066 (90.8)	3 849 (89.7)	711 (87.9)		7 626 (90.0)
Physical activity level, No. (%)				1 994 (23.5)	
i. stable high	81 (3.1)	76 (2.3)	17 (2.9)		174 (2.7)
ii. low to high	388 (14.7)	414 (12.7)	68 (11.6)		870 (13.4)
iii. high to low	91 (3.4)	109 (3.4)	21 (3.6)		221 (3.4)
iv. stable low	2 089 (78.9)	2 659 (81.6)	480 (81.9)		5 228 (80.5)
BMI^a^, mean (SD), kg/m^2^	24.0 (2.9)	25.2 (3.3)	25.9 (3.8)	40 (0.5)	24.8 (3.3)
ADI-4^a^, z-score (SD)	0.004 (3.06)	-0.036 (3.05)	0.137 (3.05)	1 377 (16.2)	-0.003 (3.06)
Smoking^a^, No. (%)				1 114 (13.1)	
i. never	1 278 (43.9)	1 780 (47.6)	378 (52.1)		3 436 (46.6)
ii. previous daily smoker	815 (28.0)	1 090 (29.2)	193 (26.6)		2 098 (28.5)
iii. daily smoker	818 (28.1)	867 (23.2)	154 (21.2)		1 839 (24.9)
Alcohol use frequency last 14 days^a^, No. (%)				1 171 (13.8)	
i. never	230 (8.0)	316 (8.5)	80 (11.2)		626 (8.6)
ii. not last 14 days	1 190 (41.2)	1 551 (41.8)	313 (43.7)		3 054 (41.7)
iii. 1–4 times	1 285 (44.5)	1 626 (43.8)	284 (39.7)		3 195 (43.7)
iv > = 5 times	184 (6.4)	218 (5.9)	39 (5.5)		441 (6.0)
Diabetes^a^, No. (%)	13 (0.4)	16 (0.4)	11 (1.4)	5 (0.1)	40 (0.5)
Blood pressure medication^a^, No. (%)	51 (1.5)	240 (5.6)	114 (14.1)	1 (0.01)	405 (4.8)
APOE ε4 carrier, No. (%)	989 (29.4)	1 285 (30.2)	252 (31.5)	60 (0.7)	2 526 (30.0)

*Abbreviations*: *BMI* body mass index (kg/m^2^), *ADI-4* anxiety and depression index

^a^From HUNT1

Table 2 shows the crude and adjusted ORs and adjusted absolute risk difference (ARD) of dementia for each group compared to the reference group. In the fully adjusted model, an overall decreasing trend in OR of dementia was observed when the groups were organized from low to high PA (OR, 0.96; 95% CI, 0.93–1.00, *P* = 0.04). We also found increased OR of dementia risk by increasing SBP within three PA groups (stable high, low to high, and stable low), while in the high to low PA group, the OR was decreasing by increasing SBP. The lowest OR of dementia was observed in the stable high PA/low SBP group (OR, 0.38; 95% CI, 0.13 to 1.10). The ARD indicated that this group was -8.34 (95% CI, -15.32 to -1.36) percentage points less likely to have dementia compared to the reference group.

**Table 2:** Odds ratio (OR) with 95% confidence interval (CI), and adjusted risk difference (ARD) with 95% CI of dementia associated with joint categories of change in physical activity (PA) and trajectories of systolic blood pressure (SBP). Based on complete case data (*n* = 6156)

	**PA and SBP groups**	**OR (95% CI)**^**a**^	**OR (95% CI)**^**b**^	**ARD % (95% CI)**^**b**^
PA stable low	SBP high	1.00 (Reference)	1.00 (Reference)	0 (Reference)
	SBP mid	0.74 (0.56, 0.97)	0.80 (0.61, 1.06)	-2.34 (-5.42, 0.75)
	SBP low	0.63 (0.47, 0.84)	0.71 (0.53, 0.96)	-3.44 (-6.64, -0.25)
PA high-low	SBP high	0.41 (0.09, 1.98)	0.49 (0.10, 2.37)	-6.53 (-18.27, 5.22)
	SBP mid	0.59 (0.29, 1.18)	0.70 (0.35, 1.41)	-3.64(-10.25, 2.98)
	SBP low	1.45 (0.78, 2.69)	1.83 (0.97, 3.45)	7.93 (-1.14, 17.01)
PA low–high	SBP high	1.21 (0.60, 2.47)	1.28 (0.62, 2.66)	3.00 (-6.16, 12.15)
	SBP mid	0.61 (0.40, 0.94)	0.73 (0.47, 1.12)	-3.30 (-7.68, 1.08)
	SBP low	0.37 (0.23, 0.62)	0.47 (0.28, 0.78)	-6.85 (-11.13, -2.58)
PA stable high	SBP high	0.56 (0.07, 4.36)	0.66 (0.08, 5.17)	-4.16 (-22.49, 14.16)
	SBP mid	0.55 (0.22, 1.35)	0.72 (0.29, 1.80)	-3.40 (-12.11, 5.32)
	SBP low	0.29 (0.10, 0.83)	0.38 (0.13, 1.10)	-8.34 (-15.32, -1.36)

^a^Adjusted for sex and age

^b^Fully adjusted

**Fig. 3:**
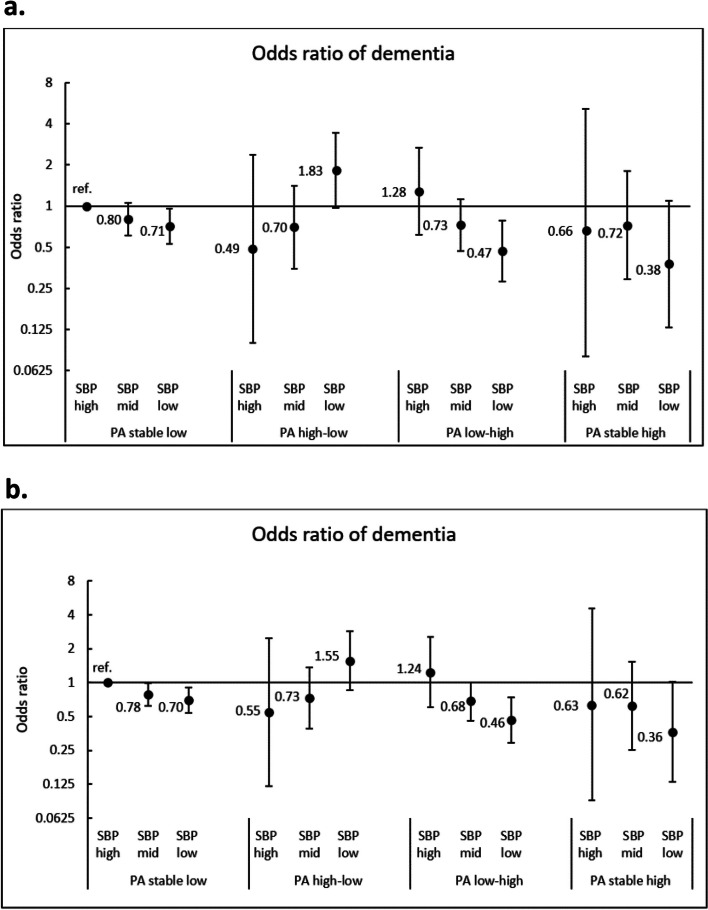
**a** Odds ratio with 95% confidence interval of dementia by physical activity (PA) and systolic blood pressure (SBP) trajectory groups. Based on complete case data (*n*=6156). **b** Odds ratio with 95% confidence interval of dementia by physical activity (PA) and systolic blood pressure (SBP) trajectory groups. Based on imputed data (*n*=8487)

In the binary logistic regression, multiple imputation gave similar pattern and estimates as complete case analysis (Fig. 3), and excluding those who reported current or previous use of BP lowering medication at HUNT1 did not change our results (Additional file 1: Appendix 4).

## Discussion

In this longitudinal cohort study with twenty-four years of follow-up, we found that individuals who stayed sufficiently active according to the WHO recommendations, and with persistent normotensive SBP in mid- to late-life had the strongest association with dementia prevention. For all PA groups, apart from those with decreasing PA level, having a lower SBP trajectory was associated with the lowest OR of dementia. The high to low PA level with increased OR of dementia by lower SBP level might be explained by reverse causation affecting PA and SBP, i.e., that prodromal dementia with degenerative neurological processes caused a decrease in PA and SBP.

The highest ARD of 8.34 percentage points between the high PA/low SBP and the reference group indicates potentially great benefits of optimizing PA and SBP levels for dementia prevention at a population level.

Our main findings of an association between high PA and low SBP trajectory in mid- to late-life and reduced dementia risk in later life is supported by other studies looking at these factors separately [10, 22, 41]. A recent meta-analysis of 243 observational prospective studies and 153 randomized controlled trials investigated evidence for Alzheimer’s disease prevention, which is the most common form of dementia [42]. They found strong evidence for hypertension in mid-life and weaker evidence for lower PA as risk factors for Alzheimer’s disease and recommend avoiding hypertension before the age of 65 and maintaining PA, especially when over 65 years old. Our finding that having a persistent SBP below 130 mm Hg was most beneficial for dementia prevention is in line with recent results from the Whitehall II cohort study where a SBP ≥ 130 mm Hg at age 50 was associated with increased risk of dementia [43]. However, most studies still focus on individual risk factors for dementia but investigating combinations of risk factors throughout the life-course has been emphasized as a need for future research [44].

### Strengths and limitations

Important strengths in our study are the large study sample with long follow-up, repeated measurements of PA and SBP, and the high quality of the diagnostic procedure of dementia [4]. We had information on many possible confounding factors at HUNT1, and also APOE genotyping, and were able to adjust for this. The overall number of missing values was low, and multiple imputation supported our results from complete case analysis.

Even if we were able to adjust for many confounding factors, there are some factors, e.g., dietary habits and social interaction, that we did not have enough information on to adjust properly for. However, we believe that this would not affect the main picture of our results. Although we did not include PA and SBP measurements from HUNT4 to minimize possible reverse causation, we cannot guarantee that our results were not affected by this. We had no cognitive assessment before HUNT4 and don’t have information on when the dementia occurred. However, we can assume that dementia cases were close to zero at HUNT1 and HUNT2 and very low at HUNT3 [45]. Including people ≥ 70 years in HUNT4 70 + makes the study prone to survival bias. It is possible that our results are statistically weaker due to PA, and SBP being related to cardiovascular disease and premature death (competing risk). Such an association was found in a previous study investigating psychological distress and dementia [46]. Possible selection bias related to participation should be noted. A previous HUNT4 70 + study investigated potential differences in participants and non-participants and found that men had lower odds for participating (not significant), decreasing odds of participation by age, and they found different odds across municipalities [4]. At HUNT3 (54% participation rate), non-participants had higher prevalence of chronic conditions, lower socioeconomic status and higher mortality but less common health issues [47]. At HUNT1 (88% participation rate) and HUNT2 (71% participation rate) only minor potential non-participation bias has been noted [47]. We did not have information about ethnicity; however, recent reports have shown that a very low proportion of the older population in this geographic area have a minority background [48]. This means that our results are mainly generalizable to a Nordic population.

We only assessed up to three SBP trajectories when modelling, due to loss of statistical power by including more groups. This means there might be other trajectories that could have made a better fit. A substantial proportion of the study sample may have a BP that to some extent is regulated by BP lowering drugs, and we cannot rule out that this may have influenced the association between BP and dementia risk. However, to avoid introducing collider bias, the main analyses were conducted without controlling for this factor. Nevertheless, the sensitivity analysis excluding people who reported use of anti-hypertensive drugs at the first survey did not change the result. To get as precise estimates as possible, PA was only retrieved from HUNT1 and HUNT3, due to a different questionnaire at HUNT2 [49]. At HUNT1, the PA recommendation was at least 20 min of vigorous activity three times a week and at HUNT3, at least 30 min each day of moderate to vigorous activity [50, 51]. This probably contributed to the low proportion of high PA, as we applied the current WHO recommendations. We made a strict cut-off on high PA and are confident that those assigned to this group meet the recommendation. However, this resulted in some small groups in the combined PA/SBP variable. It should be noted that PA were classified into two relatively broad categories, that might mask possible dose–response associations with dementia, particularly in light of evidence showing that even light PA can reduce dementia risk [7, 17]. Careful interpretation must be done considering ORs with wide CIs. This especially applies to the groups consisting of high PA.

Due to few participants in some of the PA/SBP groups we could not stratify the analyses by sex, which would have been interesting according to other literature [18, 52]. This should be investigated in future studies.

## Conclusion

Meeting the current PA recommendations from WHO in combination with persistent SBP below 130 mm Hg in mid- to late-life was associated with a reduced risk of dementia in older age compared to not meeting PA recommendations and persistent higher SBP. Our findings illustrate the need to focus on the interrelations between PA and SBP on dementia risk in clinical settings and in a life-course perspective.

### Supplementary Information

**Additional file 1: Appendix 1.** Assessment of systolic blood pressure (SBP) trajectories model fit. **Appendix 2.** Plot of individual systolic blood pressure (SBP) trajectories by age. **Appendix 3.** Distribution of dementia cases grouped by physical activity (PA) level and systolic blood pressure (SBP) trajectories. **Appendix 4.** Results from binary logistic regression after multiple imputation.

## Data Availability

Data may be obtained from a third party and are not publicly available. Data are obtained from the HUNT database (https://www.ntnu.edu/hunt).

## Abbreviations

PA: Physical activity
BP: Blood pressure
SBP: Systolic blood pressure
HUNT: The Trøndelag Health Study
WHO: World Health Organization
APOE: Apolipoprotein E
DSM-5: The Diagnostic and Statistical Manual of Mental Disorders, Fifth Edition
