# Supplementary material for: Change in physical activity and systolic blood pressure trajectories throughout mid-life and the development of dementia in older age: the HUNT study
Source: Eur Rev Aging Phys Act. 2023 Oct 2;20:18. doi: 10.1186/s11556-023-00328-1 (PMC10544393; doi:10.1186/s11556-023-00328-1)
Supplement: Supplementary file 1 — Additional file 1: Appendix 1. Assessment of systolic blood pressure (SBP) trajectories model fit. Appendix 2. Plot of individual systolic blood pressure (SBP) trajectories by age. Appendix 3. Distribution of dementia cases grouped by physical activity (PA) level and systolic blood pressure (SBP) trajectories. Appendix 4. Results from binary logistic regression after multiple imputation. [file 11556_2023_328_MOESM1_ESM.docx]

Supplemental content

Contents

[Appendix 1 2](#_Toc135734975)

[Appendix 2 2](#_Toc135734976)

[Appendix 3 3](#_Toc135734977)

[Appendix 4 3](#_Toc135734978)

Appendix 1 Assessment of systolic blood pressure (SBP) trajectories model fit.

| **SBP trajectory group** | **Average posterior group probability (%)** | **Odds of correct classification** | **Estimated probability (%) of group membership (98 % confidence interval)** | **Individuals assigned to the groups, n (%)** | **Difference in probability of group membership vs the sample proportion assigned to the group (%)** |
| --- | --- | --- | --- | --- | --- |
| Low | 82.8 | 7.3 | 40.6 (39.7-41.6) | 3383 (39.9) | 0.7 |
| Middle | 78.8 | 3.6 | 48.4 (47.6-49.3) | 4293 (50.6) | 2.2 |
| High | 81.7 | 42.1 | 10.9 (10.3-11.6) | 811 (9.6) | 1.3 |

Appendix 2 Plot of individual systolic blood pressure (SBP) trajectories by age where 1 = low, 2 = middle and 3 = high.

Appendix 3 Distribution of dementia cases grouped by physical activity (PA) level and systolic blood pressure (SBP) trajectories. Complete data on the combined PA/SBP-variable and dementia variable (n=6439).

Appendix 4 Results from binary logistic regression after multiple imputation.

|  | **PA and SBP groups** | **OR (95 % CI) of dementia^a^** | **OR (95 % CI) of dementia^b^** |
| --- | --- | --- | --- |
| PA stable low | SBP high | ref | *Ref.* |
|  | SBP mid | 0.78 (0.62-0.99) | 0.76 (0.58, 0.98) |
|  | SBP low | 0.70 (0.54-0.90) | 0.68 (0.52, 0.89) |
|  |  |  |  |
| PA high-low | SBP high | 0.55 (0.12-2.48) | 0.34 (0.05, 2.21) |
|  | SBP mid | 0.73 (0.39-1.36) | 0.78 (0.41, 1.48) |
|  | SBP low | 1.55 (0.85-2.86) | 1.53 (0.83, 2.83) |
|  |  |  |  |
| PA low-high | SBP high | 1.24 (0.60-2.56) | 1.18 (0.55, 2.52) |
|  | SBP mid | 0.68 (0.46-1.01) | 0.70 (0.47, 1.06) |
|  | SBP low | 0.46 (0.29-0.74) | 0.46 (0.28, 0.74) |
|  |  |  |  |
| PA stable high | SBP high | 0.63 (0.09-4.59) | 0.66 (0.10, 4.47) |
|  | SBP mid | 0.62 (0.25-1.53) | 0.64 (0.27, 1.54) |
|  | SBP low | 0.36 (0.13-1.02) | 0.36 (0.12, 1.05) |

^a^imputed results (n=8487)

^b^imputed results (n=8018) were those who reported current or previous use of BP medication were excluded.

Abbreviations: PA, physical activity, SBP, systolic blood pressure, BP, blood pressure, ref, reference group, OR, odds ratio, CI, confidence interval
